# Supplementary material for: Inflorescence Transcriptome Sequencing and Development of New EST-SSR Markers in Common Buckwheat (Fagopyrum esculentum)
Source: Plants (Basel). 2022 Mar 10;11(6):742. doi: 10.3390/plants11060742 (PMC8950064; doi:10.3390/plants11060742)

Fig S1. GO functional classification. *X*-axis shows GO term of the next level of the three categories, *Y*-axis shows the numbers of unigenes annotated under the terms.

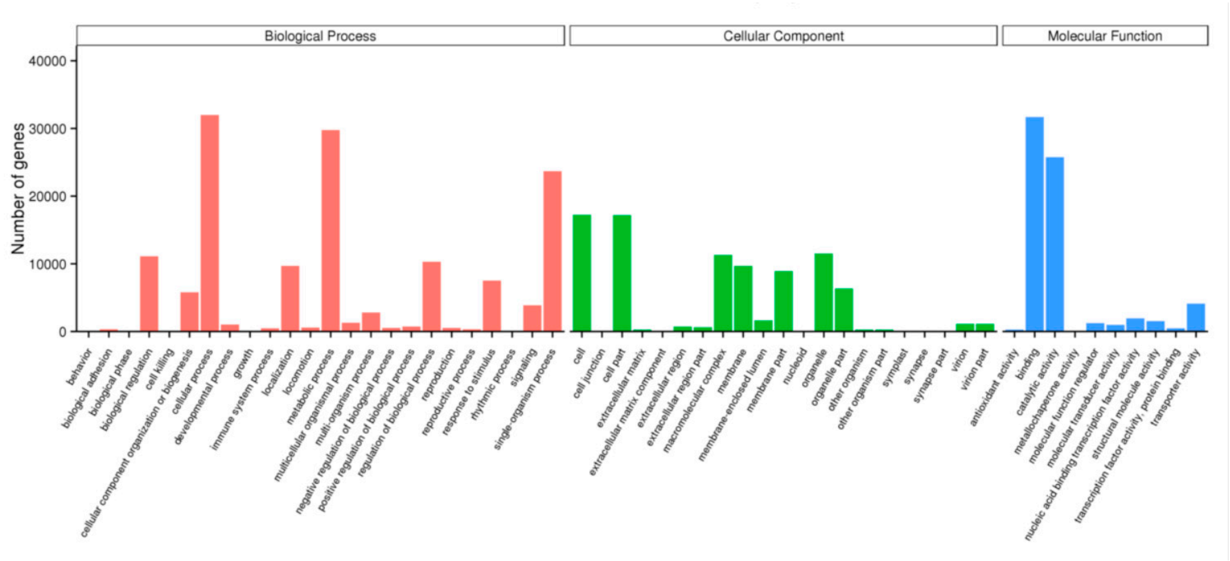

Supplement: Supplementary file 1 [file plants-11-00742-s001.zip › Fig S1.pdf]
