# Supplementary material for: Inflorescence Transcriptome Sequencing and Development of New EST-SSR Markers in Common Buckwheat (Fagopyrum esculentum)
Source: Plants (Basel). 2022 Mar 10;11(6):742. doi: 10.3390/plants11060742 (PMC8950064; doi:10.3390/plants11060742)

Fig S3. KEGG pathway assignment. *X*-axis is the number of unigenes annotated into the pathway and the proportion of the number in the total number of unigenes annotated. Y-axis is the name of KEGG metabolic pathway. (A) Cellular Processes; (B) Environmental Information Processing; (C) Genetic Information Processing; (D) Metabolism; (E) Organismal Systems

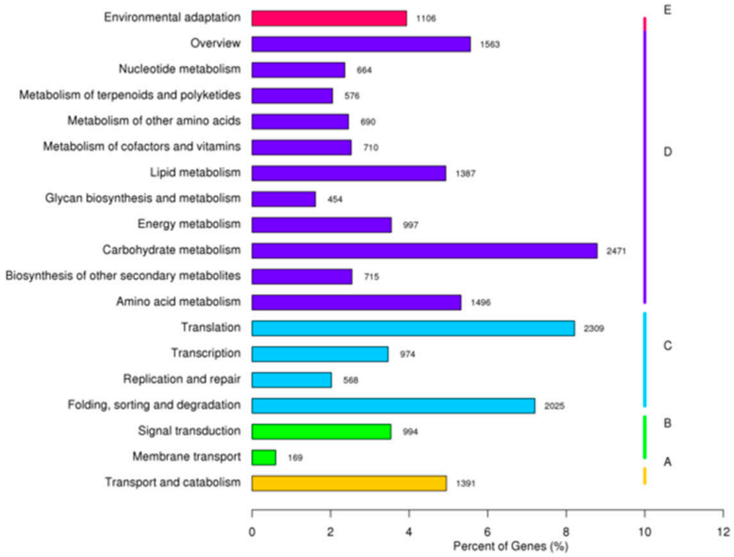

Supplement: Supplementary file 1 [file plants-11-00742-s001.zip › Fig S3.pdf]
