# Supplementary material for: Inflorescence Transcriptome Sequencing and Development of New EST-SSR Markers in Common Buckwheat (Fagopyrum esculentum)
Source: Plants (Basel). 2022 Mar 10;11(6):742. doi: 10.3390/plants11060742 (PMC8950064; doi:10.3390/plants11060742)

Fig S4. Polypropylene gel electrophoresis map for species verification of Primer SWU_Fe0156. Lane 1-35:Common buckwheat(Fe1-35); Lane36-48: Tartary buckwheat(Ft1-13). Maker:500bp

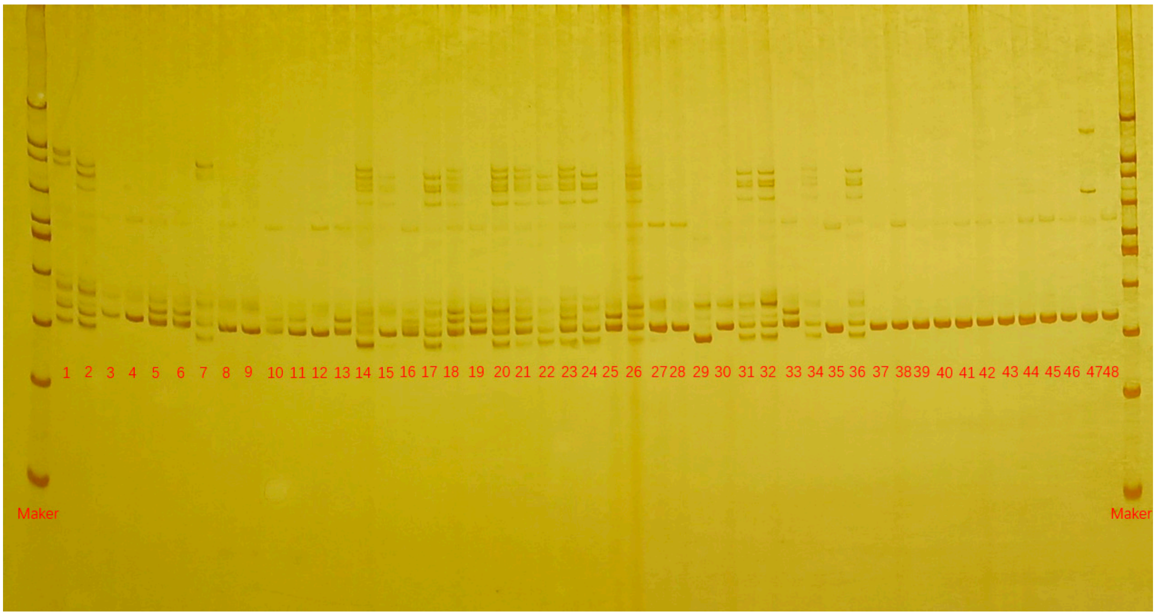

Supplement: Supplementary file 1 [file plants-11-00742-s001.zip › Fig S4.pdf]
