# Supplementary material for: Inflorescence Transcriptome Sequencing and Development of New EST-SSR Markers in Common Buckwheat (Fagopyrum esculentum)
Source: Plants (Basel). 2022 Mar 10;11(6):742. doi: 10.3390/plants11060742 (PMC8950064; doi:10.3390/plants11060742)
Supplement: Supplementary file 1 [file plants-11-00742-s001.zip › Table S1.pdf]

Table S1. A list of clean reads and reference sequence alignment.

Sample	Raw reads	Clean reads	Clean bases	Error(%)	Q20(%)	Q30(%)	GC(%)
Gr_F_1	48327536	47630570	7.14G	0.01	97.47	93.29	45.61
Gr_F_2	42364332	41788726	6.27G	0.01	97.57	93.50	45.58
UD_F_1	54440478	52870344	7.93G	0.02	97.12	92.62	45.52
UD_F_2	51885264	50344286	7.55G	0.02	97.00	92.56	45.54
