# Supplementary material for: Inflorescence Transcriptome Sequencing and Development of New EST-SSR Markers in Common Buckwheat (Fagopyrum esculentum)
Source: Plants (Basel). 2022 Mar 10;11(6):742. doi: 10.3390/plants11060742 (PMC8950064; doi:10.3390/plants11060742)
Supplement: Supplementary file 1 [file plants-11-00742-s001.zip › Table S2.pdf]

**Table S2. Summary of functional annotation of assembled unigenes.**

| | Number of Genes | Percentage(%) |
|---|---|---|
| Annotation in NR | 67950 | 57.36 |
| Annotation in NT | 43056 | 36.35 |
| Annotation in KO | 28120 | 23.74 |
| Annotation in SwissProt | 57262 | 48.34 |
| Annotation in PFAM | 54696 | 46.17 |
| Annotation in GO | 55221 | 46.62 |
| Annotation in KOG | 21928 | 18.51 |
| Annotation in all Databases | 10798 | 9.11 |
| Annotation in at least one Database | 77428 | 65.36 |
| Total Unigenes | 118448 | 100 |
